# Long-term implant integrity and complications after risk-reducing mastectomy with immediate implant reconstruction: a retrospective cohort study with prospective ultrasound follow-up at a Swedish university hospital

**DOI:** 10.1136/bmjopen-2026-124995

**Published:** 2026-10-12

**Authors:** Jessica Gahm, Marie Jaeger, Zlatan Alagic, Stina Rittri

**Affiliations:** 1 Department of Molecular Medicine and Surgery, Karolinska Institutet, Stockholm, Sweden; 2 Plastic and Reconstructive Surgery, Karolinska University Hospital, Stockholm, Sweden; 3 Victoriakliniken, Saltsjöbaden, Stockholm County, Sweden; 4 Department of Diagnostic Radiology, Karolinska University Hospital, Stockholm, Sweden; 5 Department of Clinical Science, Intervention and Technology, Karolinska Institutet, Stockholm, Sweden

**Keywords:** Ultrasound, Breast imaging, Breast surgery, Diagnostic radiology, PLASTIC & RECONSTRUCTIVE SURGERY

## Abstract

**Abstract:**

**Introduction:**

Implant-based breast reconstruction is widely performed following bilateral risk-reducing mastectomy in women at increased genetic risk of breast cancer. Although breast implants are not considered lifetime devices, there is limited evidence regarding long-term implant integrity and implant-related complications beyond 10 years after reconstruction. Current recommendations regarding long-term surveillance vary internationally, and data describing asymptomatic implant rupture and other implant-related complications in this patient population remain scarce. The aim of this study is therefore to investigate the prevalence of long-term implant-related complications in women who underwent bilateral risk-reducing mastectomy with immediate implant-based breast reconstruction.

**Methods and analysis:**

This is a retrospective cohort study with prospective clinical and ultrasound follow-up. Women who underwent bilateral risk-reducing mastectomy with immediate implant-based reconstruction at Karolinska University Hospital between 1995 and 2015 will be identified through medical records. Eligible participants are women with implants that have remained in situ for at least 10 consecutive years at the time of study inclusion.

Participants will undergo a clinical examination and standardised ultrasound assessment of implant integrity and implant-related complications. Clinical, surgical and follow-up data will be extracted from medical records. The primary outcome is the prevalence of ultrasound-detected implant rupture among women with implants in situ for more than 10 years. Secondary outcomes include capsular contracture (Baker grade III–IV), implant rotation and seroma. Patient-reported outcomes will be assessed using the BREAST-Q questionnaire. Data will be analysed using descriptive statistics, comparative analyses and multivariable logistic regression where appropriate.

**Ethics and dissemination:**

The study was approved by the Swedish Ethical Review Authority (Dnr 2026–02857-01). Participation is voluntary and requires written informed consent. Ultrasound examinations involve no ionising radiation and are associated with minimal risk. Clinically relevant findings will be managed through routine healthcare pathways. Results will be disseminated through peer-reviewed publications, conference presentations and summaries available to participants upon request.

**STRENGTHS AND LIMITATIONS OF THIS STUDY**

- Addresses a period beyond 10 years after bilateral implant-based breast reconstruction for which long-term data are scarce; inclusion of all eligible patients from a large tertiary referral centre minimises selection bias at cohort identification.
- Ultrasound assessments are performed prospectively, using predefined criteria, by plastic surgeons experienced in clinician-operated breast implant ultrasound, with confirmatory review of detected ruptures by a subspecialised breast radiologist.
- Combines objective imaging findings with clinical and patient-reported outcomes.
- Single-centre design and potential survivor bias, from the exclusion of patients with early implant-related complications, are limitations, alongside potential non-response bias among eligible patients who do not participate.
- Changes in implant types and surgical technique over the long inclusion period (1995–2015), together with reliance on historical medical records, are important limitations.

## Introduction

 Implant-based breast reconstruction is a commonly performed procedure following risk-reducing mastectomy. Well-known complications associated with implant-based reconstruction include implant rupture and capsular contracture, as well as aesthetical complications such as implant visibility or wrinkling and pain. These complications occur more frequently following implant-based breast reconstruction than after cosmetic breast augmentation, and their incidence increases over time.^1 2^ According to manufacturers and clinical guidance, silicone gel-filled implants are not lifetime devices. However, there is limited evidence to support routine implant exchange after a specific number of years, and in clinical practice, many patients retain their implants for considerably longer than the 10-year follow-up period typically reported in manufacturer studies.^3^ Because implant exchange itself carries surgical risks, routine replacement of asymptomatic implants after a predefined number of years is generally not recommended.

Rather than recommending routine implant replacement after a fixed time interval, periodic imaging surveillance has therefore been advocated to detect silent ruptures. For example, the US Food and Drug Administration recommends that patients with silicone gel-filled breast implants undergo MRI or ultrasound screening 5–6 years after implantation and every 2–3 years thereafter.^4^ Early detection of implant rupture may reduce the risk of progressive silicone leakage, capsular contracture and silicone migration to regional lymph nodes, and therefore reduce the need for more extensive revision surgery, particularly in women with implant-based breast reconstruction after mastectomy, where soft tissue coverage is often limited. Furthermore, silicone migration may cause substantial psychological stress for the patient due to suspicion of malignancy.^5–7^ In addition, peri-implant fluid collections identified during ultrasound examination may represent the rare but serious conditions breast implant-associated anaplastic large cell lymphoma (BIA-ALCL) or breast implant-associated squamous cell carcinoma.^8 9^ Despite these recommendations, previous studies have shown that many women are lost to follow-up.^10^

Many countries, including Sweden, lack specific national recommendations for long-term surveillance of breast implants, partly reflecting the scarcity of long-term follow-up studies that include imaging, such as ultrasound, beyond 10 years after implant placement in women who have undergone mastectomy and breast reconstruction. We therefore aim to investigate the prevalence of symptomatic and asymptomatic implant-related complications, together with ultrasound findings, in women who have undergone implant-based breast reconstruction and retained their implants for more than 10 years.

## Methods and analysis

This is a retrospective cohort study with prospective clinical and ultrasound follow-up, performed at the Department of Plastic and Craniofacial Surgery, Karolinska University Hospital, Stockholm, Sweden.

### Study period

Patients undergoing bilateral risk-reducing mastectomy and immediate implant-based reconstruction between 1995 and 2015 will be identified. Participant recruitment will start in June 2026 and ultrasound examinations will be performed during the second half of 2026, followed by data analysis during 2027.

### Participants

#### Inclusion criteria

- Women undergoing bilateral risk-reducing mastectomy with immediate implant-based reconstruction between 1995 and 2015 at Karolinska University Hospital.
- Ability to understand written Swedish.
- Provision of written informed consent.

#### Exclusion criteria

- Implant removal, implant exchange or conversion to autologous reconstruction within 10 years.
- Deceased patients or unreachable patients.
- Inability to participate in ultrasound examination.

### Recruitment procedures

Potentially eligible participants will be identified through hospital records and contacted by mail. Study information, consent forms and the BREAST-Q questionnaire will be provided. For non-responders, one reminder will be sent.

### Data collection

Data extracted from medical records will include demographics, smoking status, body mass index, comorbidities, operative details, implant characteristics, complications, reoperations and follow-up data.

Participants will undergo clinical examination and focused ultrasound examination of the breast implants. Capsular contracture will be assessed clinically using the Baker classification by three examining investigators, all of whom are board-certified plastic surgeons. The same three examiners will perform the ultrasound regarding implant integrity. Two of them (JG and MJ) have substantial experience in point-of-care ultrasound in implant integrity and management.^11^ Both have completed a 1-day practical training course led by a radiologist with subspecialty expertise in breast and implant ultrasonography, covering transducer handling, optimisation of image quality, recognition of relevant anatomy and identification of implant-related complications such as seroma, rupture, capsular contracture and rotation. 1 year after this initial training, both examiners were re-evaluated during a supervised session with the same radiologist, who gave direct feedback on technique, diagnostic accuracy and image quality, to confirm that their skills had been maintained. As an internal check on accuracy, an earlier study by JG and MJ found that roughly one in four examined patients subsequently underwent surgery performed by the same examiner who had carried out their ultrasound, allowing the ultrasound findings to be verified directly against intraoperative findings.^11^ The third examiner, SR, was trained afterwards by JG and MJ and, during this training period, performed at least 50 breast ultrasound examinations that were subsequently confirmed by one of the other two examiners. None of the examiners will be blinded to the patient’s symptoms or implant history, since this information is needed to guide the clinical assessment; nevertheless, every ultrasound examination will follow predefined, objective sonographic criteria, as described below, to limit subjective variation.

The ultrasound examination is focused on implant-related complications (rupture, seroma and implant rotation). Ultrasound-detected implant rupture (intracapsular and/or extracapsular), symptomatic or asymptomatic, will be defined according to established sonographic criteria, specifically a ‘snowstorm appearance’ for extracapsular rupture or a ‘stepladder sign’ for intracapsular rupture. Seroma will be defined as a clinically significant peri-implant fluid collection (>10 mL), distinct from a small amount of physiological peri-implant fluid.^12 13^ Implant rotation will be defined as any implant rotated more than 30 degrees from the implant’s vertical midline markings. Evaluation of surrounding tissues is limited to clinically relevant findings related to suspected implant rupture. In cases of an uncertain ultrasound finding, a secondary assessment will be performed by one of the other two examining plastic surgeons.

If implant rupture is detected, a confirmatory ultrasound examination will be performed by a breast radiologist (ZA) to further assess extracapsular silicone migration. In addition, if morphologically suspicious asymmetric lymph nodes without sonographic evidence of silicone deposition are detected by the breast radiologist (ZA), the most suspicious lymph node will undergo ultrasound-guided fine-needle aspiration and cytological evaluation to exclude malignancy. In cases of seroma, the patient will be referred to the standard healthcare system for ultrasound-guided fine-needle aspiration, with the aspirated fluid submitted for cytopathological evaluation for BIA-ALCL in accordance with routine clinical practice.

### Outcomes

#### Primary outcome

Prevalence of ultrasound-detected implant rupture, with ruptures classified as intracapsular or extracapsular. Implant rupture will be analysed primarily at the patient level, defined as the proportion of patients with at least one ruptured implant. Implant-level rupture rates will be reported as a supplementary analysis.

#### Secondary outcomes

Prevalence of Baker grade III–IV capsular contracture, implant rotation and seroma. Capsular contracture and seroma will be compared between ruptured and intact implants.

#### Tertiary outcome

Long-term patient-reported outcomes assessed using BREAST-Q V.2.0. BREAST-Q scores will be compared between patients with and without implant rupture. Multivariable analyses will assess the associations of implant rupture, capsular contracture, implant rotation and seroma with each BREAST-Q scale. The following BREAST-Q V.2.0 Reconstruction Module scales will be used: Psychosocial Well-being, Sexual Well-being, Satisfaction with Breasts (Postoperative), Physical Well-being (Chest), Rippling and Animation Deformity.

### Sample size calculation and statistical analysis

A formal sample-size calculation was performed for the primary objective of estimating the prevalence of ultrasound-detected implant rupture. The required sample size was calculated using the standard formula for estimation of a single population proportion^14^:

$n=\frac{Z_{1-a/2}^{2}p(1-p)}{d^{2}}$

where n is the required sample size, p is the anticipated prevalence, d is the desired absolute precision (margin of error) and $Z_{1-a/2}$ is the standard normal value corresponding to the selected CI. Based on previously reported long-term rupture rates for silicone breast implants, an anticipated implant rupture prevalence of 15% was considered reasonable for the sample-size calculation.^15 16^ Assuming an anticipated prevalence of 15% (p = 0.15), a 95% confidence level ($Z_{1-a/2}$ = 1.96) and an absolute precision of ±5 percentage points (d = 0.05), the required sample size is:


$$n=\frac{1.96^{2}\times 0.15\times 0.85}{0.05^{2}}=195.9$$

Thus, a minimum of 196 patients is required. The target sample size is rounded up to 200 patients. Assuming an anticipated participation rate of 80%, approximately 250 eligible patients (200/0.8) will therefore need to be invited to achieve the target sample size of 200 participants. This is consistent with the anticipated pool of 200–300 eligible patients identified through medical records. No finite population correction is applied as the study aims to estimate the prevalence of implant rupture in a broader target population of women who have undergone bilateral risk-reducing mastectomy with immediate implant-based breast reconstruction, rather than to estimate the prevalence exclusively within the finite cohort of eligible patients identified at the study centre.

To our knowledge, no previous study has specifically estimated the prevalence of implant rupture among asymptomatic patients whose implants have remained in situ for more than 10 years without a prior known rupture, exchange or removal. We have therefore based our assumed prevalence (15%) on the closest available estimates from studies of implant rupture in reconstruction patients with comparable long-term follow-up, while acknowledging that these estimates include both previously detected/symptomatic and newly detected/silent ruptures, and may therefore overestimate or underestimate the true prevalence of silent rupture in our more selected study population.^15 16^

Continuous variables will be summarised using means and SD or medians and IQRs, as appropriate. Categorical variables will be presented as frequencies and percentages. At the patient level, comparisons between patients with and without at least one ruptured implant will be performed using Student’s t-test or the Mann-Whitney U test for continuous variables, and the chi-square test or Fisher’s exact test for categorical variables, as appropriate. Effect estimates will be reported with 95% CIs where applicable. Multivariable linear regression analyses will be used to assess associations between implant rupture, capsular contracture, implant rotation, seroma and each selected BREAST-Q scale score. Multivariable analyses will be performed only if the number of observed complications is sufficient. For implant-level analyses, statistical methods will account for the correlation between two implants within the same patient. All statistical tests will be two-sided, and statistical significance will be defined as p<0.05. Missing data and non-responders will be handled using an available-case approach, whereby only patients or implants with evaluable data for the outcome and variables included in each analysis will contribute to the analysis. No imputation of missing data is planned. The number of observations included in each analysis and the extent of missing data will be reported.

### Patient and public involvement

Patients and members of the public were not involved in the design, conduct, reporting or dissemination plans of this study. Participants will be offered a summary of the study results on request after study completion.

### Ethics and dissemination

The study was approved by the Swedish Ethical Review Authority (Dnr 2026–02857-01). All participants will provide written informed consent before any study procedures are performed.

The study involves minimal risk. Ultrasound is non-invasive and free of ionising radiation. Any clinically relevant findings will be communicated to participants and managed through routine healthcare services.

Study findings will be disseminated through peer-reviewed publications, scientific conferences and presentations. Participants may request a summary of the study findings.

## Conclusion

This study aims to describe long-term implant integrity and implant-related complications among women who underwent risk-reducing mastectomy with immediate implant-based reconstruction and have retained their original implants for more than 10 years. As patients who underwent implant removal, exchange or conversion to autologous reconstruction within 10 years are excluded, the primary outcome reflects the prevalence of implant rupture among patients who have retained their original implants for more than 10 years and appear to be doing well with them, rather than the cumulative incidence of implant-related complications since the index operation. Given the single-centre design and the descriptive, exploratory nature of the study, the findings are expected to provide information on long-term implant integrity and implant-related complications rather than to establish definitive surveillance recommendations. The results may help inform future research and the development of surveillance strategies in this patient population.
