# Supplementary material for: Long-term implant integrity and complications after risk-reducing mastectomy with immediate implant reconstruction: a retrospective cohort study with prospective ultrasound follow-up at a Swedish university hospital
Source: BMJ Open. 2026 Oct 12;16(10):e124995. doi: 10.1136/bmjopen-2026-124995 (PMC13662027; doi:10.1136/bmjopen-2026-124995)
Supplement: Supplementary file 1 [file bmjopen-2026-124995.reviewer_comments.pdf]

PEER REVIEW HISTORY

BMJ Open publishes all reviews undertaken for accepted manuscripts. Reviewers are asked to complete a checklist review form and are provided with free text boxes to elaborate on their assessment. These free text comments are reproduced below.

ARTICLE DETAILS

Title (Provisional)

Long-term implant integrity and complications after risk-reducing mastectomy with immediate implant reconstruction: a retrospective cohort study with prospective ultrasound follow-up at a Swedish university hospital

Authors

Gahm, Jessica; Jaeger, Marie; Alagic, Zlatan; Rittri, Stina

VERSION 1 - REVIEW

**Reviewer**	**1**
**Name**	**See, Mee-Hoong**
**Affiliation**	**Universiti Malaya, Department of Surgery**
**Date**	**09-Aug-2026**
**COI**	**None**

---

1. **General / Overall**

This is a clinically relevant study protocol evaluating long-term implant outcomes after risk-reducing mastectomy and immediate implant reconstruction. The combination of medical records, clinical examination, breast ultrasound and BREAST-Q is a strength. However, several methodological issues require clarification, particularly selection bias, sample size, ultrasound assessment and statistical analysis.

2. **Title**

The title is clear but slightly broad. A more specific title could be as suggestion:

Long-term implant integrity and complications following risk-reducing mastectomy with immediate implant reconstruction: a study protocol

3. **Objective session**

The objective should be stated more clearly and consistently. The primary objective is to determine the prevalence of ultrasound-detected implant rupture after more than 10 years. Secondary objectives include capsular contracture, implant rotation, seroma and patient-

reported outcomes. The authors should clarify whether outcomes will be analysed per patient, breast or implant.

4. Methodology

Sample Size

No formal sample-size calculation was performed. The authors expect approximately 200–300 eligible patients. A formal sample-size calculation should be performed based on the primary objective: estimating the prevalence of ultrasound-detected implant rupture among women with implants in situ for more than 10 years. The authors should provide a justification showing whether this sample is sufficient to estimate implant rupture prevalence with reasonable precision. They should also estimate the expected participation rate and explain whether the final number of complications will be adequate for multivariable regression.

Methodology

The main concern is selection bias. Patients who had implant removal, exchange or conversion to autologous reconstruction within 10 years are excluded. These patients may have experienced important implant complications and excluding them may underestimate the true complication rate.

The authors should also clarify:

- How bilateral implants will be analysed.
- The ultrasound criteria for implant rupture.
- Whether the three examining surgeons will undergo standardisation.
- Whether examiners will be blinded to symptoms and implant history.
- How uncertain ultrasound findings will be confirmed.
- How missing data and non-responders will be handled.
- Which BREAST-Q modules will be used.

5. Discussion

As this is a protocol, no results discussion is required. However, the authors should include a short section discussing expected strengths and limitations. Important limitations include the single-centre design, survivor bias, non-response bias, changes in implants and surgical techniques over the long study period, and reliance on historical medical records. The current strengths and limitations section mainly lists strengths and should include the major limitations.

6. **Conclusion**

The conclusion should avoid suggesting that the study will establish definitive surveillance recommendations. It should state that the study will provide descriptive information on long-term implant integrity and complications and may help guide future research and surveillance strategies.

Final Recommendation:

Major revision. The study is important, but the authors should clarify the cohort selection, sample-size justification, ultrasound methodology, handling of bilateral implants, statistical analysis and study limitations.

VERSION 1 - AUTHOR RESPONSE

Reviewer 1

We would like to thank Reviewer 1 for their thoughtful comments. We have addressed and replied to all comments below.

Comment 1: General / Overall

This is a clinically relevant study protocol evaluating long-term implant outcomes after risk-reducing mastectomy and immediate implant reconstruction. The combination of medical records, clinical examination, breast ultrasound and BREAST-Q is a strength. However, several methodological issues require clarification, particularly selection bias, sample size, ultrasound assessment and statistical analysis.

Reply 1: Please see the changes we have made below, addressing each point in turn.

Comment 2: Title

The title is clear but slightly broad. A suggested alternative: "Long-term implant integrity and complications following risk-reducing mastectomy with immediate implant reconstruction: a study protocol".

Reply 2: We thank the reviewer and have changed the title to the suggested one: "Long-term implant integrity and complications following risk-reducing mastectomy with immediate implant reconstruction: a study protocol" (see tracked changes).

Comment 3: Objective

The objective should be stated more clearly and consistently. The primary objective is to determine the prevalence of ultrasound-detected implant rupture after more than 10 years. Secondary objectives include capsular contracture, implant rotation, seroma and patient-reported outcomes. The authors should clarify whether outcomes will be analysed per patient, breast or implant.

Reply 3: We thank the reviewer for this comment. We have clarified the unit of analysis for each outcome. Implant rupture, the primary outcome, will be analysed primarily at the patient level (i.e. the proportion of patients with at least one ruptured implant), with implant-level (per breast) rupture rates reported as a supplementary analysis. Secondary outcomes – capsular contracture, implant rotation and seroma – will be analysed at the implant (breast) level, since these can differ between the two breasts of the same patient. Because two implants from the same patient are not statistically independent, implant-level analyses will account for within-patient clustering (e.g. using cluster-robust standard errors or generalized estimating equations, as appropriate). This has been added to the Outcomes and Sample size calculation and statistical analysis sections (see tracked changes).

Comment 4: Methodology

Sample size. No formal sample-size calculation was performed. The authors expect approximately 200–300 eligible patients. A formal sample-size calculation should be performed based on the primary objective: estimating the prevalence of ultrasound-detected implant rupture among women with implants in situ for more than 10 years. The authors should provide a justification showing whether this sample is sufficient to estimate implant rupture prevalence with reasonable precision. They should also estimate the expected participation rate and explain whether the final number of complications will be adequate for multivariable regression.

Methodology. The main concern is selection bias. Patients who had implant removal, exchange or conversion to autologous reconstruction within 10 years are excluded. These patients may have experienced important implant complications and excluding them may underestimate the true complication rate.

The authors should also clarify:

• How bilateral implants will be analysed.

• The ultrasound criteria for implant rupture.

• Whether the three examining surgeons will undergo standardisation.

• Whether examiners will be blinded to symptoms and implant history.

• How uncertain ultrasound findings will be confirmed.

• How missing data and non-responders will be handled.

• Which BREAST-Q modules will be used.

Reply 4: We thank the reviewer for these important points and address each in turn.

Sample size: A formal sample-size calculation was performed for the primary objective of estimating the prevalence of ultrasound-detected implant rupture. Assuming an anticipated prevalence of 15%, a 95% confidence level and a desired precision of ±5 percentage points, a sample of 196 patients is required (rounded up to 200). Assuming a participation rate of 80%, approximately 250 eligible patients (200/0.8) will need to be invited, consistent with the anticipated pool of 200–300 eligible patients identified through medical records. To our knowledge, no previous study has specifically estimated the prevalence of implant rupture among asymptomatic patients whose implants have remained in situ for more than 10 years without a prior known rupture, exchange or removal. We have therefore based our assumed prevalence (15%) on the closest available estimates from studies of implant rupture in reconstruction patients with comparable long-term follow-up, while acknowledging that these estimates include both previously detected/symptomatic and newly detected/silent ruptures, and may therefore over- or underestimate the true prevalence of silent rupture in our more selected

study population (refs 15 and 16). This has been added to the Sample size calculation and statistical analysis section.

Selection bias: We agree that excluding patients who underwent implant removal, exchange or conversion to autologous reconstruction within 10 years may introduce selection bias and could underestimate the cumulative complication rate since the original surgery. We have added a short Conclusion section clarifying that our primary outcome should be interpreted as the prevalence of implant rupture among patients who have retained their original implants for more than 10 years – that is, among patients who appear to be doing well with their implants at this time point – rather than as the cumulative incidence of implant-related complications since the index operation. This distinction, and the term "prevalence" rather than "incidence", is also acknowledged as survivor bias in the Strengths and limitations section.

Bilateral implants: As above (Reply 3), implant rupture will be analysed primarily at the patient level, with implant-level analyses as a supplementary analysis; secondary outcomes will be analysed at the implant level, accounting for within-patient clustering. This has been specified in the Methods and analysis section.

Ultrasound criteria for implant rupture and rotation: We have specified that implant rupture will be defined according to established sonographic criteria: a "snowstorm appearance" for extracapsular rupture, or a "stepladder sign" for intracapsular rupture. Seroma will be defined as a clinically significant peri-implant fluid collection (>10 mL), distinct from a small amount of physiologic peri-implant fluid. Implant rotation will be defined as any implant rotated more than 30 degrees from the implant's vertical midline. This has been added to the Data collection section.

Standardisation and blinding of examiners: Ultrasound examinations will be performed by three examining investigators, all of whom are board-certified plastic surgeons. Two of them (J.G. and M.J.) have substantial experience in Point-of-care ultrasound (POCUS) in implant integrity and management (11). Both have completed a one-day practical training course led by a radiologist with subspecialty expertise in breast and implant ultrasonography, covering transducer handling, optimisation of image quality, recognition of relevant anatomy, and identification of implant-related complications such as seroma, rupture, capsular contracture and rotation. One year after this initial training, both examiners were re-evaluated during a supervised session with the same radiologist, who gave direct feedback on technique, diagnostic accuracy and image quality, to confirm that their skills had been maintained. As an internal check on accuracy, an earlier study by J.G. and M.J. found that roughly one in four examined patients subsequently underwent surgery performed by the same examiner who had carried out their ultrasound, allowing the ultrasound findings to be verified directly against intraoperative findings. The third examiner, S.R., was trained afterwards by J.G. and M.J. and, during this training period, performed at least 50 breast ultrasound examinations that were subsequently confirmed by one of the other two examiners. None of the examiners will be blinded to the patient's symptoms or implant history, since this information is needed to guide the clinical assessment; nevertheless, every ultrasound examination will follow predefined, objective sonographic criteria to limit subjective variation. This has been added to the Data collection section.

Confirmation of uncertain findings: In cases of an uncertain ultrasound finding, a secondary assessment will be performed by one of the other two examining plastic surgeons. If implant rupture is detected, a confirmatory ultrasound examination will be performed by a breast radiologist (Z.A.) to further assess extracapsular silicone migration. In addition, if the breast radiologist detects morphologically suspicious asymmetric lymph nodes without sonographic evidence of silicone deposition, the most suspicious lymph node will undergo ultrasound-guided fine-needle aspiration and cytological evaluation to exclude malignancy. In cases of seroma, the patient will be referred to the standard healthcare system for ultrasound-guided fine-needle aspiration, with the aspirated fluid submitted for cytopathological evaluation for BIA-ALCL, in accordance with routine clinical practice. This has been clarified in the Data collection section.

Missing data and non-responders: Missing data and non-responders will be handled using a complete-case (available-case) analysis; only patients or implants with evaluable data for a given outcome will be included in the corresponding analysis. No imputation of missing data is planned. This has been specified in the Sample size calculation and statistical analysis section, and non-response is acknowledged as a limitation.

BREAST-Q modules: The following BREAST-Q Version 2.0 Reconstruction Module scales will be used: Psychosocial Well-being, Sexual Well-being, Satisfaction with Breasts (Postoperative), Physical Well-being (Chest), Rippling, and Animation Deformity. This has been specified in the Outcomes / Data collection section.

Comment 5: Discussion

As this is a protocol, no results discussion is required. However, the authors should include a short section discussing expected strengths and limitations. Important limitations include the single-centre design, survivor bias, non-response bias, changes in implants and surgical techniques over the long study period, and reliance on historical medical records. The current strengths and limitations section mainly lists strengths and should include the major limitations.

Reply 5: We agree and have expanded the Strengths and limitations section to include the following limitations: the single-centre design; potential survivor bias, since patients who experienced early implant-related complications leading to implant removal, exchange or autologous conversion are excluded; potential non-response bias among eligible patients who do not participate; changes in implant types and surgical techniques over the long inclusion period (1995–2015); and reliance on historical medical records, which may be incomplete for some variables (see tracked changes).

Comment 6: Conclusion

The conclusion should avoid suggesting that the study will establish definitive surveillance recommendations. It should state that the study will provide descriptive information on long-term implant integrity and complications and may help guide future research and surveillance strategies.

Reply 6: We agree and have added a short Conclusion section (placed after the Ethics and dissemination section) which states that the study will provide descriptive information on long-term implant integrity and implant-related complications, and may help inform future research and surveillance strategies, rather than establishing definitive surveillance recommendations, given the single-centre design and descriptive, exploratory nature of the study (see tracked changes).

Once again, we are grateful for the opportunity to revise our manuscript, and we hope the Editors and Reviewer find our changes satisfactory.
